# Colonel Waring

**Published:** 1899-06

**Authors:** 


### COLONEL WARING.

If this city (New York) owes a lasting debt of gratitude to Colonel George E. Waring, Jr., for his energetic and most efficient service in his sanitary work in New York, he has placed the nation under a still more lasting obligation for his work in Cuba, which should be met as far as is possible by an Act of Congress granting a pension to his family. Holding a commission in the civil service, Colonel Waring's death would not be included in the pension list of the army and navy, but who in the army and navy has done better work or encountered greater peril? The commission of which Colonel Waring was the head was sent to Havana to devise some plan to improve its sanitary condition, and thereby rid the Western Continent of one of its worst breeding places of yellow fever. In the prosecution of his work Colonel Waring was indefatigable, visiting without hesitation the most poison-tainted localities in the city, and formulating a plan which will undoubtedly be adopted by government, which will rid the island of a pestilence which has not only been its scourge for hundreds of years, keeping back its progress, but a menace to the health of the adjoining islands and the United States. The public gratitude for the work Colonel Waring has accomplished, and in the prosecution of which he lost his life, should be met by some more substantial reward than votes of thanks, or monuments of bronze or marble. We look to Congress for an act which will place the family of the hero in a position above pecuniary want.—*Medical Times.*

